# The Diagnostic Value of Laryngeal Paralyses

**Published:** 1905-06-24

**Authors:** 


### THE DIAGNOSTIC VALUE OF LARYNGEAL PARALYSES.

In a lecture on this subject Mr. Harold Barwell[1] emphasises the importance of the laryngoscope in medical practice as affording information not available by any other method and of great significance in diagnosis. In particular, in this respect, is it important to bear in mind that some forms of laryngeal paralysis may exist without any appreciable disturbance of the voice function. Thus these may in individual cases be entirely overlooked unless a laryngoscopic examination is made. A paralysis due to a lesion of the superior laryngeal nerve is uncommon though the nerve may be damaged by a wound, as in cut throat, or by the pressure of a tumour. The paralysis is limited to one muscle—namely, the crico-thyroid. This is, of course, a tensor of the vocal cord, and when it is paralysed the cord will remain "slack" on phonation, and will "flap" during respiration. But to a large extent these defects are concealed by the action of the other laryngeal muscles, and hence the paralysis may be overlooked even with the aid of the laryngoscope. It is in anæsthesia of the larynx that a lesion of the superior laryngeal nerve finds its most marked expression. The most frequent and conspicuous laryngeal paralyses occur in the area of the recurrent laryngeal nerves which supply all the muscles except the crico-thyroid. The muscles in any gradually progressive lesion of the nerve path invariably fail in a definite order. The paralysis is first seen in the abductor (posterior crico - arytenoid) muscle, next in the internal tensor, and last in the adductor (lateral crico-arytenoid). Abductor paralysis is evidenced mainly by the fixation of the affected cord in the middle line. As the normal cord meets it in phonation there is no interference with the voice. But as in inspiration the affected cord is not carried outwards the available aperture of the glottis is only one - half the normal size, and thus there may be dyspnœa on active exertion. When the abductor paralysis is bilateral both cords lie close to the middle line. On inspiration they are not abducted, and, indeed, their edges are forced close together by the pressure of the inrushing air. Dyspnœa with inspiratory stridor is a marked symptom, and the liability to paroxysmal exacerbations makes the condition so dangerous a one that tracheotomy is a wise precaution. When the internal tensor (thyro-arytenoid) fails the edge of the cord is no longer straight, but forms a concave line, and later, as the adductor muscle is paralysed, the cord passes into the cadaveric position, that is, midway between the middle line and the normal position of rest. Should the paralysis affect both sides both cords will occupy the cadaveric position and the voice will be lost. In all these cases certain other details regarding the position of the arytenoid cartilages may also be observed.

It is necessary to distinguish a true laryngeal palsy from fixation of the cord due to local disease, such as syphilis, tubercle, perichondritis, etc. In these conditions scars, swelling, or deformity can usually be observed.

The phonatory (adduction) function of the larynx is controlled by centres in the cerebral cortex, and hence in functional (hysterical) disease the laryngeal paralysis is always an adductor one. A unilateral cerebral lesion does not produce laryngeal paralysis, just as it does not produce paralysis of the muscles of respiration. But a bilateral lesion affecting both tracts may cause a bilateral adductor paralysis, though such a lesion is very uncommon. Cases of so-called " functional aphonia " (bilateral adductor paralysis) are common in hysteria, but in many, perhaps in the majority of instances they are not purely hysterical. Anything which increases the effort of phonation—

constitutional debility, thickening of the cords, or laryngeal catarrh—may prevent the close and accurate apposition of the cords in phonation. In pure hysteria cough is usually not aphonic, and the onset and recovery are alike sudden. The voice in many cases is promptly regained on the application of a faradic currrent or a strong astringent. In all these instances of bilateral adductor paralysis the paralysis is seldom complete. Such a paralysis promptly produces aphonia, which at once attracts attention, and in this respect the cases contrast with cases of paralysis affecting the abductors. They are not due to serious organic disease, and are not in themselves dangerous to life.

Organic lesions in the medulla or at the base of the brain, in the vagus, or the recurrent laryngeal nerve produce abductor paralysis. In bulbar or basal lesions nuclei in the neighbourhood of those governing the muscles of the larynx are apt to be affected, and hence paralyses may also be present in the soft palate, uvula, and pharynx. Paralysis of one cord and of the same side of the palate is an important indication of nuclear disease. Persistent frequency of the pulse from involvement of the cardio-inhibitory centre is another sign of bulbar disease. For the most part nuclear paralyses are bilateral, but there are exceptions to this rule. Tabes dorsalis is the commonest cause. The paralysis may be the only clinical evidence of the disease. Other laryngeal symptoms in tabes are anæsthesia, paræsthesia, and "laryngeal crises." General paralysis is another not uncommon cause of abductor paralysis. Specific disease affecting the nuclei, gummata at the base of brain, and pachymeningitis are responsible for many cases of laryngeal palsy; in such instances the sixth nerve is also often paralysed. In bulbar paralysis laryngeal palsy (bilateral) is the rule, but it is usually late in the course of the disease. Among rare causes syringo-myelia and amyotrophic lateral sclerosis may be mentioned. Disseminated sclerosis rarely produces paralysis, but intention tremors may be well seen in the larynx. Of peripheral causes it is necessary to recognise toxic influences, as lead, alcohol, and arsenic; also infections, as diphtheria and, less frequently, typhoid fever and influenza. Possibly tubercle and syphilis sometimes act in this way. And a transient paralysis may perhaps be produced by "cold." Gross peripheral lesions usually affect the vagus, below the level of the superior laryngeal nerve, or the recurrent laryngeal nerve, and are rarely bilateral. The most frequent causes are aneurysm, enlarged glands, usually tuberculous, and cancer of the œsophagus. Less common are mediastinal growths, pulmonary tubercle, goitre, cancer of the lung, pleurisy, and massive pericardial effusion. In aneurysm the paralysis is commonly on the left side, owing to the relation of the recurrent nerve to the aorta. An aneurysm of the innominate may involve the right nerve, and with a very large aneurysm, or in consequence of the existence of two aneurysms, the paralysis may be bilateral. It should be remembered that paralysis of the left cord may be the only sign of thoracic aneurysm. In addition to paralysis an aneurysm may cause spasm of the glottis through irritation of the vagus or trachea. The same irritation is responsible for cough which usually has a "brassy" quality, though when the paralysis is complete the cough becomes "wheezy" like the cough of a cow. Tuberculosis may produce recurrent paralysis either by pressure of bronchial glands, when the left cord is generally affected, or by involvement of the nerve in a tuberculous infiltration at the apex of the lung. In the latter circumstances paralysis is commonly right-sided, as the right nerve lies close to the pleura. Bilateral recurrent paralysis may be due either to cancer of the œsophagus, to enlargement of the thyroid (simple or malignant), or, as already mentioned, to a very large thoracic aneurysm.

[1] Lancet, June 3,1905.

